# Holistic approach to assess the association between the synergistic effect of physical activity, exposure to greenspace, and fruits and vegetable intake on health and wellbeing: Cross-sectional analysis of UK Biobank

**DOI:** 10.3389/fpubh.2022.886608

**Published:** 2022-09-30

**Authors:** Catalina Cruz-Piedrahita, Charlotte J. Roscoe, Caroline Howe, Daniela Fecht, Audrey de Nazelle

**Affiliations:** ^1^Centre for Environmental Policy, Imperial College London, London, United Kingdom; ^2^Harvard T.H. Chan School of Public Health, Harvard University, Boston, MA, United States; ^3^MRC Centre for Environment and Health, School of Public Health, Imperial College London, London, United Kingdom

**Keywords:** health and wellbeing, synergies, lifestyle behaviors, greenspace, physical activity

## Abstract

**Background:**

Urban agriculture has been shown to contribute to healthy lifestyle behaviors, such as increased fruit and vegetable intake and greater exposure to greenspaces and there is plenty of evidence linking these lifestyle behaviors to better health and wellbeing. However, most evidence relates to assessing one behavior at a time despite available epidemiological research showing how the combined effects of multiple behaviors are associated with health and wellbeing. This research aims to examine the association of the interactions between various lifestyle behaviors and exposures related to urban agriculture and health and wellbeing.

**Methods:**

We used data from the UK Biobank baseline questionnaire (*N*~500, 000) to assess the association of two lifestyle behaviors (fruit and vegetable intake and physical activity) and greenspace exposure, with four health and wellbeing markers (blood pressure, BMI, self-health assessment, and self-reported loneliness) independently, and in combination. Associations between lifestyle behaviors, greenspace exposure, and the possible interactions with health and wellbeing were explored using general linear models (GLMs), adjusted for socio-demographic confounders including age, sex, educational qualifications, index of multiple deprivation, and ethnicity, and a lifestyle confounder: smoking status.

**Results:**

After removing missing data, as well as participants who did not meet the inclusion criteria, the final study sample was *n* = 204,478. The results indicate that meeting recommended levels of the World Health Organization (WHO) for fruits and vegetable intake, and the advice from the UK Chief Medical Officer for physical activity, is linked to better health and wellbeing markers. We found that UK Biobank participants who lived in greener areas and were physically active were more likely to feel alone and think their health was poor. Participants who were physically active and met the recommended intake of fruits and vegetables were more likely to have healthy blood pressure, feel less lonely, and rate their health as good. Evidence of three-way interactions was weak, and mostly was not associated with the health and wellbeing markers assessed here.

**Conclusion:**

Taken in combination, healthy diets, physical activity and exposure to greenspaces are associated with health and wellbeing. In some cases, these effects are synergistic, indicating associations above and beyond the mere additive effect of the behaviors considered independently. Promoting such behaviors together, for example, through urban agriculture, is therefore more likely to generate greater public health changes than if they are promoted through independent policies and programs. Inter-relationships between these pathways and different health and wellbeing markers, however, are complex, and require further investigation to understand optimal environments and conditions for urban health promotion.

## Introduction

With a growing global population, increasingly agglomerated within urban centers (1), we are faced with new challenges, such as the lack of access to healthy food sources, fewer recreational areas and places to exercise and be in contact with nature (2, 3). These issues have been linked to an increase in obesity rates (4, 5), chronic diseases, mental health issues, and feelings of isolation (4, 6, 7). The average consumption of fruits and vegetables in countries of the Global North is lower than the recommended levels. For example, only 29% of British adults eat at least five portions of fruits and vegetables, whilst the average consumption is 3.8 portions (8). Moreover, Cobiac et al. (9) showed that interventions to encourage change in dietary behavior, such as counseling or worksite promotion, are neither effective nor cost-efficient in increasing fruit and vegetable consumption. Similarly, Guthold et al. (10) assessed population trends from 2001 to 2016 and found that insufficient levels of physical activity are still on the rise worldwide. Despite the well-established understanding of the importance of being physically active, having a healthy diet with at least five portions of fruits and vegetables, and of having access to greenspace in cities, health authorities and city planners are still struggling to find public interventions that facilitate such healthy behaviors. A solution, such as urban agriculture, could potentially promote these healthy lifestyles simultaneously (physical activity, healthy diets, and access to greenspace), making such urban policy solutions particularly attractive.

Several authors have proposed a combined effect of healthy diets and exercise behaviors on the prevalence and prevention of non-hereditary diseases and mental health conditions. One longitudinal study by Pierce et al. (11) found that the combination of having a healthy diet and exercising was associated with a significant increase in the survival rate of women with breast cancer. A cross-sectional study in the US by Loprinzi et al. (12) found that people who have healthy diets and are physically active scored better on cardiovascular biomarkers than people who only undertook one or neither of these health behaviors. People with unhealthy dietary behaviors and low levels of exercise were 2.4 times more likely to have metabolic syndrome than the comparison group. In another paper, the same authors, Loprinzi and Mahoney (13) showed that, in addition to independent associations of such behaviors, people with two and three of the healthy lifestyle behaviors were 67 and 82% less likely of suffering symptoms of depression, respectively, compared to those with none of the behaviors. Although these studies demonstrate a cluster relationship between diet and exercise they do not investigate the possible added association of the synergistic effects of these behaviors with health and wellbeing markers.

Interaction effects have been shown for the combination of greenspace exposure and exercise, also known as green exercise. One cross-sectional experimental study found that running on a treadmill whilst watching pleasant natural landscapes reduced participants' blood pressure, while watching unpleasant urban environments increased the diastolic blood pressure (14). A meta-analysis of 143 studies investigating more than 100 health markers related to exercising outdoors found that walking or running outdoors was more positively associated with health markers than the same activity undertaken indoors, as measured by systolic and diastolic blood pressure, heart rate, body fat percentage, BMI, cholesterol levels, maximal oxygen consumption (VO2 max), depression, and physical functionality (15).

To our knowledge, no research has evaluated the combined effects between food intake and green spaces, nor the possible synergistic associations between fruit and vegetable intake, physical activity, and greenspace exposure with health. Nevertheless, Pretty et al. (16) proposed a theoretical framework describing the pathways and interactions between diet, physical activity, connections to nature and communities and physical health and wellbeing. In the paper, the author reported urban agriculture projects as a type of green exercise with the added outcome of food production (16). Research indicates that urban agriculture projects could promote these behaviors and exposures together (17).

The Food and Agriculture Organization of the United Nations (FAO) defines urban agriculture as: “the growing of plants and the raising of animals for food and other uses within and around cities and towns, and related activities such as the production and delivery of inputs, processing, and marketing of products” (18). The concept and focus of research into urban agriculture varies between countries in the Global South and Global North (19). In the latter, urban agriculture is linked to environmental and social movements (20, 21), its potential to improve public health (22), and to address urban problems related to environmental injustice and food security (23–25). Urban agriculture movements have become very popular amongst citizens as a recreational activity, a source of healthy food provision, or a place to escape from the city environment (26, 27). In 2013 in the UK, waiting lists for allotments totalled 86,787 people; in many cases, the waiting period for an allotment can last years (28). Moreover, cities in the Global North have included urban agriculture as a public health strategy due to its association with mental health, physical health, and wellbeing (29–31). Urban agriculture has also been correlated with a reduction in body mass index (BMI) and anxiety (32, 33), and increased self-esteem and wellbeing (33–35). Wood et al. (36) found that participation in allotments was associated with improvement in mood, self-esteem, and general health, and a reduction of depression and fatigue. The authors argue that allotments were associated with wellbeing because of green exercise and healthy eating. However, most of the associations between related urban agriculture's lifestyle behaviors and positive associations with health and wellbeing have been investigated in isolation (33), and the evidence of a multi-behavior association between health and wellbeing markers and lifestyle behaviors is based on anecdotal evidence.

This paper aims to explore synergistic associations on health and wellbeing of urban agriculture-related behaviors and exposures (physical activity, fruit and vegetable intake, and greenspace exposures). We hypothesize that diet, physical activity, and greenspace exposure are synergistically related to good health and wellbeing markers. Nevertheless, assessing these possible synergies relies on a large sample, and so far, to our knowledge, no such sample of urban gardeners exists. However, these lifestyle behaviors and synergies are not exclusive to urban agriculture; hence it is possible to assess the synergies within another population sample; we used the UK Biobank cohort for this purpose.

## Methods

A cross-sectional analysis was conducted on data obtained from the UK Biobank baseline assessment to explore the potential synergistic effect of the lifestyle behaviors; (1) fruit and vegetable intake; (2) physical activity; and (3) exposure to greenspace on health and wellbeing markers.

### UK Biobank cohort

The UK Biobank cohort is a sample of the British population containing health and lifestyle information for around 500,000 people between 40 and 69 years old at recruitment between 2006 and 2010. Possible participants were identified using the NHS patient registers living near an assessment center (~25 miles or ~40 Km) across England, Wales, and Scotland. The invitations to participate were sent *via* post. In total, 9.2 million invitations were sent, and 503,000 people agreed to participate (37). At the assessment centers, participants completed a touchscreen questionnaire answering questions related to socio-demographics, lifestyle, environmental, and health-related factors (37, 38). Participants were also verbally interviewed and gave urine, blood, and saliva samples. UK Biobank's protocol, and procedures are reported in detail elsewhere (39, 40). For this research, we only included participants who resided in England at the time they were recruited and had data collected *via* the touchscreen questionnaire, direct measurements at the assessment center, and had greenspace data available from UK Biobank project 4362, which assigned high-resolution Ordnance Survey MasterMap greenspace data in circular distance buffers around participant addresses (*N* = 204,478) (For the project's permits see the section “ethics approval and consent to participate” below).

### Measures

The explanatory variables and health and wellbeing markers were selected *a priori* due to their previously assessed relationship with urban agriculture projects, and their relevance to public health (41–44).

### Explanatory variables

The variable fruit and vegetable intake was created by adding the portions of fresh fruits, raw vegetables, and cooked vegetables obtained from three questions from the touchscreen questionnaire: (1) “On average, how many heaped tablespoons of COOKED vegetables would you eat per DAY? (Do not include potatoes; put ‘0' if you do not eat any),” (2) “On average, how many heaped tablespoons of SALAD or RAW vegetables would you eat per DAY? (Include lettuce and tomato in sandwiches; put ‘0' if you do not eat any),” and (3) “About how many pieces of FRESH fruit would you eat per DAY? (Count one apple, one banana, 10 grapes etc. as one piece; put ‘0' if you do not eat any).” The three questions have the same instruction for the participants (Please provide an average considering your intake over the last year. If you are unsure, please provide an estimate or select Do not know), and the same answer options (“Enter a number/Less than one/Do not know/Prefer not to answer”). If participants answered, “Less than one,” we replaced it for a value of 0.5 (45), and we excluded participants who answered “Do not know,” or “Prefer not to answer.” Based on the NHS values for fruit and vegetable portions, three heaped tablespoons of cooked vegetable and four heaped tablespoons equal one portion, consequently, before creating the variable fruit and vegetable intake, we divided cooked vegetable intake by three, and raw vegetable intake by four. Then we used the WHO recommendation of eating at least five portions of fruits and vegetables to categorize the variable; we labeled participants as “Low fruit and vegetable intake” for participants who ate less than five portions a day and “High fruit and vegetable intake” for those who ate five or more portions a day.

To assess physical activity, questions based on the short form of the International Physical Activity Questionnaire (IPAQ) were used (46), the protocol and procedures are reported elsewhere (47). Physical activity is reported in weekly Metabolic Equivalent Tasks (METs) (min/week), which relates to the energy expenditure doing moderate activities, vigorous activities, and walking, compared to sitting (46, 47). We used the UK Biobank variable “IPAQ activity group,” which categorizes people into three groups, “Low,” “Moderate,” and “High,” by using the classification processing rules published by the IPAQ (46).

Based on our hypothesis that people who practice urban agriculture are in constant proximity and interaction with nature, we wanted to assess the association of greenspace exposure and health and wellbeing markers in using a close distance. Hence, we assessed exposure to greenspace within 100 m from a participant's residential address using data from Ordnance Survey MasterMap Greenspace (UK Biobank project 4362). Methods to calculate greenspace percentage are reported elsewhere (48). The percentage of surrounding greenness or greenspace exposure was categorized using quartiles (Q1, Q2, Q3, and Q4).

### Sociodemographic characteristics and lifestyle factors

Covariates were selected based on assumptions that they would confound the relationship between the exposure and outcome, and/or were important risk factors identified by other UK Biobank researchers for the health or wellbeing markers under investigation (49–56). We included sex (Female, Male), age at baseline assessment, ethnicity as a binary variable (two categories): (1) White, (2) Not white (original Biobank categories: “Mixed,” “Asian or Asian British,” “Black or Black British,” “Chinese,” and “Other ethnic group”), highest educational qualification (four categories): (1) College or University Degree; (2) Other professional qualification or education to age 18 (original Biobank categories: “A levels/AS levels or equivalent,” “NVQ or HND or HNC or equivalent,” and “Higher National Certificate (HNC) or equivalent, other professional qualifications”); (3) High School Qualifications (original Biobank categories: “CSEs or equivalent” and “O levels/GCSEs or equivalent”); (4) None of the above.

We defined neighborhood deprivation using the deciles of the index of multiple deprivation 2010 for England (57). We created the categories Low, Medium, and High Deprivation by collapsing the national deciles 1–3, 4–7, and 8–10, respectively. The index of multiple deprivation comprises assessments of seven different categories experienced by people: Income Deprivation, Employment Deprivation, Health Deprivation and Disability, Education Skills and Training Deprivation, Barriers to Housing and Services, Living Environment Deprivation, and Crime (58). We used the category “Smoking status” from the UK biobank that splits participants into current smokers, previous smokers, and participants who had never smoked.

### Health and wellbeing markers

We defined health markers BMI (three categories) by collapsing the original UK Biobank variable: (1) Healthy (≤ 24.9), (2) overweight (25–29.9), and obese (≥30) (59), blood pressure (binary): (1) Healthy (≤120 systolic; ≻ 80 diastolic) and (2) High (≥120 systolic; ≥80 diastolic) (60, 61), self-health assessment, and loneliness were considered as outcome variables to assess health and wellbeing. The questions “Do you often feel lonely?” (No = 0, Yes = 1) and “How often are you able to confide in someone close to you?” (0 = almost daily to once every few months; 1 = never, or almost never), participants were defined as lonely if they answered positively to both questions (score 2) (62, 63). Participants' self-health assessment was assessed using the question “In general how would you rate your overall health?” (Answer options: Poor/Fair/Good/Excellent/Do not know/Prefer not to answer). Participants who answered “Do not know” or “Prefer not to answer” were excluded as these responses are not clearly interpretable outcomes.

### Statistical analysis

All the statistical analyses were carried out using the R software (64). Out of the initial 502,664 UK Biobank participants, we excluded participants with missing data in any of the explanatory variables and those who did not meet the inclusion criteria. To avoid losing data we classified NAs of confounders as “Missing” and used this as a category. To explore if healthy behaviors and exposures related to urban agriculture (fruit and vegetable intake, physical activity, and greenspace exposure) have a synergistic effect on health traits, we conducted Bootstrap General Linear Models (GLMs with bootstrap) with and without interactions (two-way and three-way) between the explanatory variables. We used logistic regression for blood pressure (reference = healthy) and loneliness (reference = no), and we used ordinal logistic regression to assess overall health rating (reference = poor) and BMI (reference = normal BMI), the variable overall health rating is the only variable that uses the unhealthy category (poor) as reference (62). All models were adjusted for age, sex, index of multiple deprivation, ethnicity, highest qualification, and smoking status. For each health and wellbeing marker we first fitted one single exposure model with one of the explanatory variables plus the covariates; secondly, we fitted a multi-exposure confounding model with the three explanatory variables—fruit and vegetable intake, physical activity, and greenspace exposure, plus the covariates without including interactions; finally, we fitted a multi-exposure model with two and three-levels interactions between the explanatory variables plus the covariates. To account for multiple testing, p values were corrected using the Benjamini & Hochberg method, setting the significance level at α= 0.05 and using two-tailed tests (65). To calculate the fit of the model and the 95% confidence intervals of the models' coefficients we bootstrapped all the models (1,000 generations) (66, 67). The tables in the main text show the outcomes of the exposure model with two and three-levels interactions; outcomes for single exposure and the multi-exposure confounding models are presented in Supplementary material 1. We used likelihood ratio test to compare the goodness of fit between the models with interactions and the models without interactions and statistical significance was defined by a two-side alpha level of *p* = 0.05. Models with interactions included have a better goodness of fit than those considering the health behaviors independently.

## Results

The final sample size was *n* = 204,478. Women represented 53% of the participants in the sample (*n* = 109,339). The sample was biased toward white people, who represented 93% of the sample (*n* = 190,872), and non-smokers (89%; *n* = 182,514). The percentage of participants meeting the recommendations for fruits and vegetables was 31.8% (*n* = 64,999), and 40.5% (*n* = 82,655) of the participants were classified in the high physical activity category. The percentage of participants classified with healthy BMI was 34% (*n* = 69,538), 4.0% (*n* = 8,132) of participants rated their health as poor, 83% (*n* = 169,802) had healthy blood pressure, and 4.7% (*n* = 9,624) were classified as lonely (Supplementary Table 2).

### Association between lifestyle behaviors and health and wellbeing

Single exp models show, as would be expected that both fruit and Vegetable intake and physical activity are positively associated with overall health rating; physical activity with decreased BMI; both physical activity and fruit and vegetable intake with decreased blood pressure, and physical activity and surrounding greenness with reduced loneliness. Contrary to expectations, however, fruit and Vegetable intake and surrounding greenness both were associated with increased BMI; the quartiles Q2 and Q3 of surrounding greenness with poor health, and with increased blood pressure.

After adjusting for age, sex, ethnicity, highest educational qualifications, smoking status, and Index of Multiple Deprivation with single exposure models show, as would be expected that both fruit and Vegetable intake and physical activity are positively associated with overall health rating; physical activity with decreased BMI; both physical activity and fruit and vegetable intake with decreased blood pressure, but the latter only for high levels of physical activity (OR = 0.894, 95% CI = 0.865, 0.924, *p* adjusted ≤ 0.001; Supplementary Table 5). Physical activity and surrounding greenness were associated with reduced loneliness, except for the 2nd quartile of greenspace exposure that was not statistically significant (OR = 0.979, 95% CI = 0.924, 1.038, *p* adjusted = 0.477; Supplementary Table 6). Contrary to expectations, however, fruit and vegetable intake and surrounding greenness were associated with increased BMI; the quartiles Q2 and Q3 of surrounding greenness with poor health, and with increased blood pressure.

We found largely similar associations between the explanatory variables and the health outcomes in the multiple-exposure models with no interactions, but all the association were slightly attenuated when compared to the single exposure models (Supplementary Table 5).

Multiple-exposure models with two- and three-way interactions show generally similar associations between the explanatory variables and the health outcomes, but with further attenuation. More closely aligned with expectations, Q2 (Q2: OR = 1.049, 95% CI = 0.984, 1.118, *p* adjusted = 0.249) and Q4 (Q4: OR = 1.049, 95% CI = 0.907, 1.030, *p* adjusted = 0.372) were no longer significantly associated with increased BMI, but Q3 in comparison with Q1 remained associated with worsened BMI (Table 1). Also more aligned with expectations, the quartiles Q2 and Q3 were no longer associated with poor health, and in reverse, as would be expected, Q4 became significantly associated with good health (OR = 0.896, 95% CI = 0.825, 0.974, *p* adjusted ≤ 0.032). More aligned with expectation the greenspace exposure quartiles Q2 and Q4 lost their positive associations with blood pressure, but Q3 remained significant (OR = 1.199, 95% CI = 1.097, 1.311, *p* adjusted ≤ 0.001; Table 1). Physical activity and greenspace exposure remained significant, showing a beneficial association with loneliness (Table 1).

**Table 1:** Odds Ratio (OR) with 95% bootstrap confidence intervals (bCIs), and Benjamini & Hochberg adjusted *p*-value of greenspace exposure at a 100 m buffer [GE; reference = (Greenspace Q 1)], physical activity levels (IPAQ; reference = Low), fruits and vegetables intake (FyV; reference = <5 portions), and the interactions between these variables for BMI (reference = Healthy), overall health rating (reference = Poor), blood pressure (reference = Healthy), loneliness (reference = No).

**Exposure variable (increment)**	**OR**	**95% CI**	***p*-adjusted**
**Body Max Index (BMI) (reference = Healthy)**			
**Fruit and vegetable intake (reference= Does not meet fruit and vegetable recommendation)**			
Meets fruit and vegetable	1.169	1.062, 1.287	0.003
**Greenspace exposure; 100 m buffet (reference = quartile 1)**			
Greenspace Q2	1.049	0.984, 1.118	0.249
Greenspace Q3	1.077	1.010, 1.149	0.047
Greenspace Q4	0.967	0.907, 1.030	0.372
**Physical activity levels; IPAQ (reference = Low)**			
Moderate physical activity	0.639	0.604, 0.675	< 0.001
High physical activity	0.509	0.480, 0.538	< 0.001
**Interaction between fruit and vegetable and greenspace**			
Meets fruit and vegetable; Greenspace Q2	1.005	0.880, 1.148	0.973
Meets fruit and vegetable; Greenspace Q3	1.031	0.903, 1.178	0.738
Meets fruit and vegetable; Greenspace Q4	0.998	0.874, 1.139	0.975
**Interaction between fruit and vegetable and physical activity**			
Meets fruit and vegetable; Moderate physical activity	0.937	0.838, 1.047	0.335
Meets fruit and vegetable; High physical activity	0.851	0.763, 0.950	0.009
*Interaction between greenspace and physical activity*			
Greenspace Q2; Moderate physical activity	1.056	0.977, 1.141	0.280
Greenspace Q3: Moderate physical activity	1.046	0.968, 1.130	0.335
Greenspace Q4: Moderate physical activity	1.071	0.992, 1.157	0.146
Greenspace Q2; High physical activity	1.049	0.969, 1.135	0.335
Greenspace Q3: High physical activity	1.023	0.945, 1.108	0.689
Greenspace Q4: High physical activity	1.077	0.995, 1.166	0.130
**Interaction between fruit and vegetable intake, greenspace and physical activity**			
Meets fruit and vegetable; Greenspace Q2; Moderate physical activity	1.018	0.871, 1.189	0.898
Meets fruit and vegetable; Greenspace Q3: Moderate physical activity	1.005	0.861, 1.175	0.973
Meets fruit and vegetable; Greenspace Q4: Moderate physical activity	1.037	0.888, 1.211	0.738
Meets fruit and vegetable; Greenspace Q2; High physical activity	1.108	0.950, 1.292	0.303
Meets fruit and vegetable; Greenspace Q3: High physical activity	1.096	0.941, 1.278	0.335
Meets fruit and vegetable; Greenspace Q4: High physical activity	1.099	0.943, 1.280	0.335
**Overall health rating (reference = Poor)**			
**Fruit and vegetable intake (reference= Does not meet fruit and vegetable recommendation)**			
Meets fruit and vegetable	1.030	0.932, 1.138	0.637
**Greenspace exposure; 100 m buffet (reference = quartile 1)**			
Greenspace Q2	0.980	0.917, 1.046	0.629
Greenspace Q3	1.006	0.941, 1.074	0.869
Greenspace Q4	1.140	1.067, 1.218	< 0.001
**Physical activity levels; IPAQ (reference = Low)**			
Moderate physical activity	1.801	1.700, 1.907	< 0.001
High physical activity	3.009	2.835, 3.193	< 0.001
**Interaction between fruit and vegetable and greenspace**			
Meets fruit and vegetable; Greenspace Q2	1.135	0.988, 1.303	0.122
Meets fruit and vegetable; Greenspace Q3	1.028	0.895, 1.180	0.728
Meets fruit and vegetable; Greenspace Q4	1.068	0.929, 1.228	0.425
**Interaction between fruit and vegetable and physical activity**			
Meets fruit and vegetable; Moderate physical activity	1.166	1.038, 1.309	0.019
Meets fruit and vegetable; High physical activity	1.183	1.055, 1.327	0.008
**Interaction between greenspace and physical activity**			
Greenspace Q2; Moderate physical activity	0.985	0.909, 1.067	0.728
Greenspace Q3: Moderate physical activity	0.980	0.905, 1.062	0.682
Greenspace Q4: Moderate physical activity	0.951	0.878, 1.031	0.308
Greenspace Q2; High physical activity	0.903	0.832, 0.981	0.030
Greenspace Q3: High physical activity	0.920	0.847, 0.999	0.041
Greenspace Q4: High physical activity	0.842	0.775, 0.915	< 0.001
**Interaction between fruit and vegetable intake, greenspace and physical activity**			
Meets fruit and vegetable; Greenspace Q2; Moderate physical activity	0.839	0.713, 0.987	0.048
Meets fruit and vegetable; Greenspace Q3: Moderate physical activity	0.918	0.781, 1.080	0.377
Meets fruit and vegetable; Greenspace Q4: Moderate physical activity	0.864	0.734, 1.017	0.124
Meets fruit and vegetable; Greenspace Q2; High physical activity	0.897	0.764, 1.053	0.264
Meets fruit and vegetable; Greenspace Q3: High physical activity	0.916	0.780, 1.074	0.360
Meets fruit and vegetable; Greenspace Q4: High physical activity	0.880	0.749, 1.034	0.179
**Blood pressure (reference = Healthy)**			
**Fruit and vegetable intake (reference= Does not meet fruit and vegetable recommendation)**			
Meets fruit and vegetable	1.095	0.958, 1.253	0.241
**Greenspace exposure; 100 m buffet (reference = quartile 1)**			
Greenspace Q2	1.088	0.994, 1.190	0.120
Greenspace Q3	1.199	1.097, 1.311	< 0.001
Greenspace Q4	1.045	0.954, 1.145	0.388
**Physical activity levels; IPAQ (reference = Low)**			
Moderate physical activity	1.023	0.945, 1.108	0.603
High physical activity	0.896	0.825, 0.974	0.032
**Interaction between fruit and vegetable and greenspace**			
Meets fruit and vegetable; Greenspace Q2	0.830	0.687, 1.003	0.114
Meets fruit and vegetable; Greenspace Q3	0.850	0.706, 1.024	0.134
Meets fruit and vegetable; Greenspace Q4	0.884	0.732, 1.068	0.252
**Interaction between fruit and vegetable and physical activity**			
Meets fruit and vegetable; Moderate physical activity	0.835	0.713, 0.979	0.067
Meets fruit and vegetable; High physical activity	0.862	0.737, 1.008	0.120
**Interaction between greenspace and physical activity**			
Greenspace Q2; Moderate physical activity	0.964	0.864, 1.076	0.562
Greenspace Q3: Moderate physical activity	0.912	0.818, 1.016	0.142
Greenspace Q4: Moderate physical activity	0.974	0.873, 1.087	0.654
Greenspace Q2; High physical activity	1.062	0.949, 1.189	0.358
Greenspace Q3: High physical activity	0.926	0.828, 1.036	0.241
Greenspace Q4: High physical activity	1.026	0.916, 1.150	0.654
**Interaction between fruit and vegetable intake, greenspace and physical activity**			
Meets fruit and vegetable; Greenspace Q2; Moderate physical activity	1.248	0.999, 1.559	0.114
Meets fruit and vegetable; Greenspace Q3: Moderate physical activity	1.317	1.058, 1.639	0.043
Meets fruit and vegetable; Greenspace Q4: Moderate physical activity	1.226	0.982, 1.532	0.123
Meets fruit and vegetable; Greenspace Q2; High physical activity	1.187	0.953, 1.479	0.179
Meets fruit and vegetable; Greenspace Q3: High physical activity	1.211	0.975, 1.505	0.134
Meets fruit and vegetable; Greenspace Q4: High physical activity	1.275	1.023, 1.589	0.074
**Loneliness (reference = No)**			
**Fruit and vegetable intake (reference= Does not meet fruit and vegetable recommendation)**			
Meets fruit and vegetable	1.161	0.966, 1.394	0.150
**Greenspace exposure; 100 m buffet (reference = quartile 1)**			
Greenspace Q2	0.952	0.836, 1.085	0.525
Greenspace Q3	0.858	0.749, 0.983	0.047
Greenspace Q4	0.736	0.638, 0.849	< 0.001
**Physical activity levels; IPAQ (reference = Low)**			
Moderate physical activity	0.687	0.611, 0.772	< 0.001
High physical activity	0.679	0.602, 0.766	< 0.001
**Interaction between fruit and vegetable and greenspace**			
Meets fruit and vegetable; Greenspace Q2	0.729	0.552, 0.963	0.047
Meets fruit and vegetable; Greenspace Q3	0.799	0.602, 1.062	0.160
Meets fruit and vegetable; Greenspace Q4	0.911	0.679, 1.222	0.567
**Interaction between fruit and vegetable and physical activity**			
Meets fruit and vegetable; Moderate physical activity	0.746	0.590, 0.943	0.030
Meets fruit and vegetable; High physical activity	0.695	0.553, 0.874	0.004
**Interaction between greenspace and physical activity**			
Greenspace Q2; Moderate physical activity	1.068	0.902, 1.264	0.523
Greenspace Q3: Moderate physical activity	1.134	0.953, 1.349	0.196
Greenspace Q4: Moderate physical activity	1.283	1.071, 1.537	0.016
Greenspace Q2; High physical activity	0.995	0.837, 1.183	0.957
Greenspace Q3: High physical activity	1.059	0.886, 1.266	0.567
Greenspace Q4: High physical activity	1.181	0.980, 1.423	0.120
**Interaction between fruit and vegetable intake, greenspace and physical activity**			
Meets fruit and vegetable; Greenspace Q2; Moderate physical activity	1.402	0.989, 1.988	0.089
Meets fruit and vegetable; Greenspace Q3: Moderate physical activity	1.253	0.877, 1.791	0.262
Meets fruit and vegetable; Greenspace Q4: Moderate physical activity	1.042	0.720, 1.507	0.854
Meets fruit and vegetable; Greenspace Q2; High physical activity	1.489	1.057, 2.096	0.046
Meets fruit and vegetable; Greenspace Q3: High physical activity	1.468	1.037, 2.078	0.049
Meets fruit and vegetable; Greenspace Q4: High physical activity	1.367	0.955, 1.955	0.124

All models were adjusted for sex, age, highest educational qualification, ethnicity, Index of Multiple Deprivation, and smoking status,label = tblr:interactions, notea = Cells in gray were significant.

#### Two-way interactions

Our results show that the synergistic effect of eating at least five portions of fruits and vegetables whilst being physically active is associated with healthy blood pressure, that is; after controlling for the association between each of the lifestyle variables independently and blood pressure, the effect of the interaction term in the category “meets the recommended levels of fruit and vegetable” is larger among the individuals with “high” physical activity (OR = 0.851, 95% CI = 0.763, 0.950, *p* adjusted = 0.009) when compared to those individuals with low levels of physical activity, or vice versa.

Moreover, the effect of the interaction term in the category “meets the recommended levels of fruit and vegetable” and the categories: moderate and high physical activity levels are larger and negatively associated with health marker loneliness (OR = 0.746, 95% CI = 0.590, 0.943, *p* adjusted = 0.030) and (OR = 0.695, 95% CI = 0.553, 0.874, *p* adjusted = 0.004), respectively. We observed the same outcome for BMI, but in this case, the interaction term was dose-response, meaning that people who meet the required intake of fruit and vegetable and have “high” levels of physical activity have lower odds of being overweight or obese (OR = 1.166, 95% CI = 1.038, 1.309, *p* adjusted = 0.019) than those with “moderate” physical activity (OR = 1.183, 95% CI = 1.055, 1.327, *p* adjusted = 0.008), respectively.

Although unexpected, after controlling for the association between each of the lifestyle variables independently and overall health rating, the effect of the interaction term in the categories Q1, Q2, and Q3 of greenspace shows an extra negative association only for individuals with “high” physical activity levels; (OR = 0.903, 95% CI = 0.832, 0.981, *p* adjusted = 0.030), (OR = 0.920, 95% CI = 0.847, 0.999, *p* adjusted = 0.041), and (OR = 0.842, 95% CI = 0.775, 0.915, *p* adjusted ≤ 0.001), respectively.

The effect on the interaction term in the category “moderate” physical activity and the fourth quartile of greenspace exposure was large and associated with loneliness (OR = 1.283, 95% CI = 1.071, 1.537, *p* adjusted = 0.016). That is, people with moderate levels of exercise that live in areas with high percentage of greenness have higher are more prone to feel alone.

#### Three-way interactions

The interaction between the three explanatory variables was generally not associated with the health markers. Still, we found that the effect on the interaction term in the categories meeting the recommended intake of fruits and vegetables, moderate physical activity, and the Q2 of greenspace was large and negatively associated with overall health rating (OR = 0.839, 95% CI = 0.713, 0.987, *p* adjusted = 0.048). This means that people who meet the recommended intake of fruit and vegetable, exercise moderately and live in a slightly greener area than the reference group are more likely to report their health as poor than those in the reference groups.

Participants “meeting the recommended intake of fruits and vegetable,” that exercise “moderately,” and their homes are located in a third quartile of greenspace were more likely to have high blood pressure when compared to the reference groups (OR = 1.317, 95% CI = 1.058, 1.639, *p* adjusted = 0.043). The effect on the interaction term in the categories “meeting the recommended intake of fruits and vegetables,” “moderate” physical activity, and the “Q2 of greenspace” was large and negatively associated with overall health rating (OR = 0.839, 95% CI = 0.713, 0.987, *p* adjusted = 0.048).

The effect on the interaction term in the category “high” physical activity, the “second” and “fourth” quartiles of greenspace exposure and the category “meeting the recommended intake of fruits and vegetables was large and positively associated with loneliness (OR = 1.489, 95% CI = 1.057, 2.096, *p* adjusted = 0.046), and (OR = 1.468, 95% CI = 1.037, 2.078, *p* adjusted = 0.049), respectively. That is, participants with high levels of exercise living in a greener area than the reference group and eating at least five portions of fruit and vegetables are more likely to feel alone than the reference groups.

## Discussion

Through an analysis of a large sample of the UK population; the UK Biobank, we aimed to explore the potential synergistic effects of health pathways resulting from the practice of urban agriculture. This study is novel in that it looks at the independent, and combined associations of two lifestyle behaviors (fruit and vegetable intake and physical activity), and greenspace exposure on different health and wellbeing markers (blood pressure, BMI, self-reported loneliness, and overall health rating) whilst taking advantage of a large population sample (the UK Biobank).

### Main associations between lifestyle behaviors and health and wellbeing markers

Numerous epidemiological studies have shown associations between unhealthy lifestyle behaviors and life-threatening chronic diseases (68–70). Our current analysis shows that, after controlling for confounders, physical activity and fruit and vegetable intake show positive associations on most health and wellbeing markers. This is in-line with a large body of literature that demonstrates that being physically active reduces the chances of suffering from chronic diseases including various forms of cancer, high blood pressure problems, cholesterol, cardiorespiratory conditions, mental illnesses, bone and muscular problems, obesity, and sleep complications (71–74). Insufficient intake of fruit and vegetables has been shown to cause 31% of ischemic heart diseases, approximately 19% of gastrointestinal cancers, and 11% of stroke (75–77) whilst increased intake of vegetables on the other hand significantly reduces the risk of cardiovascular incidents (75). Contrary to expectations, however, meeting fruit and vegetable intake recommendations was shown to increase BMI in our analyses. A possible reason for this result is that for our sample, a high intake of fruit and vegetable might reflect a high intake of food in general, and research shows the size of portions is directly linked with high BMI (78).

In our single exposure models, although greenspace is shown to be associated with reduced loneliness, it was negative associated on health as measured by BMI, blood pressure, and overall health rating. Whilst the statistical significance of these associations is mostly removed in the multi-exposure and interaction models, this is still contrary to evidence from the literature. Greenspace exposure has been shown to be positively associated with mental health benefits (79–81), reduction in cardiovascular morbidity (82), type II diabetes (83, 84), and mortality (85). The reason for this observation in this study could be due to the 100 m distance buffer we used to measure greenspace exposure, whilst as most research identifies benefits for buffers of 300 m or more. For example, similar to our results, Zhang et al. (86) found a negative association between the percentage of green space and depression symptoms in adolescents at a 100 m buffer in adjusted models and no association once the models were corrected for confounders. Hartley et al. (87) found no significant association between the percentage of green space and several mental issues in kids and adolescents at a 200 m buffer. Still, they found a negative association between greenspace and anxiety for buffer distances at 400 and 800 m. Moreover, it has been shown that the benefits of natural spaces to people are not one-dimensional and depend on several factors, such as proximity to the green area, type of greenspace, how often the greenspace is visited, and the time spent in the greenspace (88–90). Coleman et al. (91) for instance demonstrated how air pollution may form part of the equation in the relationships between exercise, greenspace, and mortality. Therefore, further research that includes information on the type of green space and distance is needed. Another possible explanation for that outcome is the presence of a confusing variable not assessed here, such as working status and conditions, marital status and the quality of family relationships (92–95).

#### Interactions

Beyond the independent associations of healthy lifestyles and health and wellbeing markers, our study demonstrated further combined associations of healthy physical activity levels, diet, and greenspace exposure with health and wellbeing markers. Models with interactions included showed a better fit to the data than the models that considered the lifestyle behaviors independently. This indicates that the lifestyle behaviors are most likely to act in combination with one another, rather than alone.

#### Fruit and vegetable intake and physical activity

Our results show that the synergistic effect of eating at least five portions of fruits and vegetables whilst being physically active is associated with healthy BMI, a good overall health rating, and lower feelings of loneliness, that is; after controlling for the association between each of the lifestyle variables independently and BMI, overall health rating, and loneliness, the effect of the interaction term in the category “meets the recommended levels of fruit and vegetable” is larger among the individuals that belong to categories of moderate and high physical activity when compared to those individuals with low levels of physical activity, or vice versa. Previous studies have also shown that people who improve their diet, and concurrently increase their levels of physical activity, lose on average more weight than expected than if they had undertaken the behaviors independently (96). Evidence suggests that synergies between diet and physical activity have also been shown to lower levels of blood pressure, cardiovascular diseases, depression, and certain cancers (12, 96–98). However, contrary to this evidence, we could not find evidence supporting a synergistic effect on blood pressure of eating at least five portions of fruits and vegetables whilst being physically active. Probably, other aspects of diet are needed besides just the intake of fruit and vegetable, for example portions size, or sugar and saturate fat intake (99, 100).

#### Physical activity and greenspace exposure

Surprisingly, we found negative synergies between greenspace exposure and high levels of exercise on participants reporting good health. Moderate levels of physical activity and exposure to the highest quartile of greenspace also led to higher odds of feeling alone. None of the other interactions with greenspace were statistically significant. These results contradict previous findings on added benefits for health and wellbeing of green exercise (101), although our metrics precluded any assessment of where physical activity took place or of how respondents were engaged in greenspace. Other research, however, has found little or no evidence of physical activity being a mediator of green space benefits (4, 53, 91, 102–104). Lahart et al. (105) carried out a meta-analysis of the benefits for health and wellbeing of exercising outdoors versus indoors and reported inconclusive, poorly supported, and contradictory findings overall. Whilst some studies have shown clear benefits from green exercise, lack of conclusiveness in this area may indicate that relationships are complex, and evaluations might be limited by poor standardization of measures of green space exposure. Our results suggest that despite the proposed association between green exercise and physical health and wellbeing, the association is not always positive and greener areas are not necessarily associate with higher levels of physical activity and better health and wellbeing.

#### Three-way interactions

In general, the three-way interactions show little or no association with the health and wellbeing markers, and for some specific combinations, indicate potential negative impacts. This is likely to be caused by the dominant negative impact of greenspace on outcomes in our analysis. For example, the interaction between fruits and vegetable intake and physical activity showed a clear positive association with good health rating, but, when greenspace is also considered, the interaction between these three lifestyle behaviors with overall good health when physical activity is moderate and greenspace exposure is moderate to low, is negative. Feelings of loneliness showed the same trend, the interaction between fruit and vegetable and physical activity was associated with lower odds of feeling alone, but, when greenspace is also included the interaction between the three lifestyle behaviors was associated with higher odds of feeling alone. However, three-way interactions are complex, and the significant associations need to be considered with care. Further analysis needs to corroborate the nature and significance of these types of interactions (106).

Previous studies indicate the importance of other forms of interactions or synergies, which we were not able to investigate. This suggests that it is likely that multiple factors within a person's environment act together to affect their health. For instance, healthy diets and vegetable intake have been shown to attenuate the detrimental impacts of air pollution (107), or vise versa, high levels of air pollution have been shown to attenuate some of the benefits of physical activity (108). Some synergies show a dose-response relationship between multiple healthy behaviors and health and wellbeing markers (13, 98), whilst for others such as greenspace the association is not dose-response (53). This research thus supports a growing body of research that demonstrates how the urban environment contributes in multiple ways to public health (109, 110). It also shows how more research focused on holistic approaches that consider the multiple pathways between urban environment, health and wellbeing is needed. The extra association effects of synergies occurring between different health behaviors are clearly complex; simplistic approaches to public health policies will therefore not necessarily take advantage of positive associations and may, in fact, have unintended adverse effects. It is therefore recommended that a holistic approach to public health be taken rather than targeting one behavior individually as has been carried out to date in both research and policy planning in this area.

### Interactions and urban agriculture

Returning to our hypothesis that interventions such as urban agriculture can bring more significant benefits to public health than interventions that only seek to address a single healthy lifestyle behavior: as stated in the introduction, research has shown the benefits of urban agriculture for health and wellbeing and the association between the practice of urban agriculture and healthy lifestyles (33). Our results continue to support the existing literature that multiple-lifestyle behaviors are better than single-focused approaches (111). However, more research is needed to understand the association that the different interactions between the urban environment, sociodemographic factors, and lifestyle behaviors have with health and wellbeing. Nevertheless, and more importantly, there is a need for health authorities to create strategies that motivate the population to engage in healthy behaviors (112). According to our results and the literature, urban agriculture could be a helpful tool due its multiple potential contributions to urban health (113).

## Limitations

The strength of this study is the large sample size and number of lifestyle behaviors and health markers assessed. However, the study presents several limitations. We could not establish causality due to the cross-sectional nature of this study, so we cannot discard the possibility of reverse causality (114). Secondly, the UK Biobank had a response rate of only 5% of the people invited to participate, low response rates are prone to bias (115) and it in this case the UK Biobank cohort was biased toward healthy participants, so it cannot be considered as a population estimate for several of the health markers (49, 116). Moreover, self-reported data is known to lead to social desirability bias (117–120), whereby people tend to over-report traits in questionnaires, patterns of this behavior have been reported in dietary data, physical activity, self-health assessment and wellbeing markers (117–120), hence, it is possible that in our sample fruit and vegetable intake, physical activity, loneliness, and overall health rating were prone to desirability bias. Fourthly, we only used some information to assess diet and greenspace exposure, and we know these lifestyle behaviors have more components. For the diet variable, we only used the fruit and vegetable intake. Other aspects of the diet affect health and wellbeing, such as intake of processed foods, sugar, saturated fats, fibre intake, and even the amount of food consumed per day (121–125). We only considered the percentage of green coverage for greenspace, but evidence has shown that the quality of greenspace also affects health and wellbeing (126). Also, it is not the same to live close to greenspace as being able to access the greenspace itself (127, 128). Moreover, we only used the information of greenspace coverage around the households, and we did not have information of other places where people might spend time, such as workplaces.

We acknowledge the issue of extrapolating populations (129). Different populations might have different lifestyle behaviors and might be subject to different environmental pressures. But we believe that the UK Biobank cohort is close enough to the population that practices urban agriculture to be able to make the extrapolation. Finally, our models did not include other important determinants of health and wellbeing, such as genetics (130).

## Conclusion

Despite the widespread and known benefits of healthy lifestyles, it remains a challenge to encourage the general population to take many of these behaviors up, particularly in tandem. The WHO estimates that 1.7 million lives could be saved globally by increasing the consumption of fruit and vegetables (131); Saint-Maurice et al. (132) show that adding 10 min of moderate or vigorous exercise could prevent around 110,000 deaths per year in the US. Residential greenness has been shown to lower mortality risk in most of the causes of death analyzed (133). Public health gains from these lifestyle factors are undeniable, yet little progress is made on the ground to increase uptakes. Solutions that enable seamless integration of healthy habits in daily lives are needed, and, as we see from this research, solutions that enable the simultaneous uptake of healthy habits are likely to achieve even greater health changes than tackling each of these behavior or exposure target one at a time. Physical activity, fruit and vegetable intake, and exposure to greenspace, are two lifestyle behaviors and one exposure that can be achieved jointly through participation in urban gardening. This research provides some of the arguments for the promotion of urban agriculture that go beyond the known independent associations that have been previously identified in relation to urban agriculture participation and health. Nevertheless, we can also see from our research how associations between lifestyle behaviors and health and wellbeing are complex and not necessarily associated as expected. More multi-behaviors research is needed to increase the evidence around the association between lifestyle behaviors combined and health and wellbeing markers.

## Data availability statement

The data analyzed in this study is subject to the following licenses/restrictions: The UK Biobank owns the data used for this study, restrictions to the availability of these data apply. The data were used under license for this research. Requests to access these datasets should be directed to: https://www.ukbiobank.ac.uk/.

## Ethics statement

The studies involving human participants were reviewed and approved by the North West Multi-Centre Research Ethics Committee, the Scottish Community Health Index Advisory Group, and the Wales Patient Information Advisory Group. The patients/participants provided their written informed consent to participate in this study.

## Author contributions

CC-P and CR performed data cleaning, interpretation, and statistical analysis from the dataset. CC-P wrote the first draft. CR contributed to the study design and manuscript correction. AN, CH, and DF contributed to the study design and manuscript revision. All authors have read and approved the final version of this paper.

## Funding

This work was supported by the MRC Centre for Environment and Health, which is currently funded by the Medical Research Council (MR/S019669/1, 2019-2024). Infrastructure support for the Department of Epidemiology and Biostatistics was provided by the NIHR Imperial Biomedical Research Centre. CC-P was funded by the Colombian Administrative Department of Science, Technology, and Innovation (COLCIENCIAS) during the realization of this project.

## Conflict of interest

The authors declare that the research was conducted in the absence of any commercial or financial relationships that could be construed as a potential conflict of interest.

## Publisher's note

All claims expressed in this article are solely those of the authors and do not necessarily represent those of their affiliated organizations, or those of the publisher, the editors and the reviewers. Any product that may be evaluated in this article, or claim that may be made by its manufacturer, is not guaranteed or endorsed by the publisher.
