# Supplementary material for: Holistic approach to assess the association between the synergistic effect of physical activity, exposure to greenspace, and fruits and vegetable intake on health and wellbeing: Cross-sectional analysis of UK Biobank
Source: Front Public Health. 2022 Sep 30;10:886608. doi: 10.3389/fpubh.2022.886608 (PMC9561552; doi:10.3389/fpubh.2022.886608)
Supplement: Supplementary file 1 [file Data_Sheet_1.PDF]

Holistic approach to assess the combined associations physical activity, exposure to greenspace, and fruits and vegetable intake on health and well-being: Cross-sectional analysis of UK Biobank- Supplementary material

Catalina Cruz-Piedrahita[1], Charlotte J. Roscoe[2,3], Caroline Howe[1], Daniela Fecht[3], and Audrey de Nazelle[1]

[1]Centre for environmental policy. Imperial College London

[2]Harvard T.H. Chan School of Public Health, Harvard University, Landmark Center, 401 Park Drive, Boston, MA, 02215, USA

[3]MRC Centre for Environment and Health, School of Public Health, Imperial College London, Norfolk Place, London, W2 1PG, UK

UK Biobank data fields

Table 1: UK Biobank data field of the variables used in this study. The data field is the number given to the variable in the UK Biobank. Data can be access at: https://biobank.ndph.ox.ac.uk/ukb/browse.cgi

Variable name	UK Biobank data field
**Sociodemographics**	

Continued on next page

(Continued)

Variable name	UK Biobank data field
Sex	31
Ethnicity background	21000
Index of Multiple Deprivation (small-area level)	26410
Age when attended assessment centre	21003
Qualifications	21003
**Lifestyle factors**	
Smoking status	20116
**Lifestyle behaviours related to urban agriculture**	
Cooked vegetable intake	1289
Salad / raw vegetable intake	1299
Fresh fruit intake	1309
IPAQ activity group	22032
Greenspace percentage, buffer 300m	24503
**Health and well-being indicators**	
Loneliness; (reference = No), isolation Mental health	2020
Overall health rating; (reference = No)	2178
Diastolic blood pressure, automated reading	4079
Systolic blood pressure, automated reading	4080
Seen doctor (GP) for nerves, anxiety, tension or depression; (reference = No)	2090
Waist circumference	48
Hip circumference	49

[a] Some variables are further process or were used to create new variables for the present study, the methods are described in the main text.

Sociodemographics of the UK Biobank sample used in this research

Table 2: Data field of the UK Biobank variables used in this study

Characteristic	n = 204,478
**Age at baseline (years)**	56 (8)
**Sex**	
Female	109,339 (53%)
Male	95,139 (47%)
**Ethnicity, binary**	
Not white	13,606 (6.7%)
White	190,872 (93%)
**Index of Multiple Deprivation (small-area level)**	
Low deprivation	77,366 (38%)
Medium deprivation	79,564 (39%)
High deprivation	47,548 (23%)
**Highest qualification**	
None of the above	30,139 (15%)
education till 16	55,142 (28%)
18 above no degree	36,938 (19%)
College or University degree	72,301 (37%)
**Smoking status**	
Never	110,908 (54%)
Previous	71,606 (35%)

Continued on next page

(Continued)

Characteristic	n = 204,478
Current	21,469 (11%)
**Body Max Index (BMI)**	
Healthy	69,538 (34%)
Overweight	86,588 (42%)
Obese	48,352 (24%)
**Blood pressure**	
Healthy	169,802 (83%)
High	34,676 (17%)
**Loneliness**	
No	194,854 (95.3%)
Yes	9,624 (4.7%)
**Overall health status**	
Poor	8,132 (4.0%)
Fair	41,826 (20%)
Good	119,884 (59%)
Excellent	34,636 (17%)
**Greenspace (%) at a 100 m buffer; quartiles**	
1	51,156 (25%)
2	51,093 (25%)
3	51,140 (25%)
4	51,089 (25%)
**Physical activity; IPAQ activity group**	

Continued on next page

(Continued)

Characteristic	n = 204,478
Low	37,967 (18.5%)
Moderate	83,856 (41%)
High	82,655 (40.5%)
**Fruits and vegetables intake, binary**	
less than 5 portions a day	139,479 (68,2%)
5 or more portions a day	64,999 (31.8%)

Model 1: Single exposure models

Body Max Index (BMI)

Table 3: Single association between physical activity, fruit and vegetable intake, and greenspace exposure and BMI. Categories were Healthy, overweight, and Obese (reference = Healthy).

Exposure variable (increment)	OR	95% CI	p adjusted
**Physical activity (reference= Healthy)**			
Moderate	0.662	0.647, 0.678	< 0.001*
High	0.522	0.510, 0.535	< 0.001*
**Fruit and vegetable intake (reference= less than 5 portionss)**			
High	1.040	1.022, 1.059	< 0.001*
**Greenspace exposure in quartiles (reference= Quartile 1)**			
Quartile 2	1.126	1.100, 1.154	< 0.001*
Quartile 3	1.146	1.118, 1.174	< 0.001*
Quartile 4	1.057	1.031, 1.083	< 0.001*

Overall health rating

Table 4: Single association between physical activity, fruit and vegetable intake, and greenspace exposure and overall health rating. Categories were Poor, Fair, Good, and Excellent (reference = Poor).

Exposure variable (increment)	OR	95% CI	p adjusted
**Physical activity (reference= Healthy)**			
Moderate	1.795	1.751, 1.840	< 0.001*
High	2.877	2.805, 2.950	< 0.001*
**Fruit and vegetable intake (reference= less than 5 portions)**			
5 or more portions of fruit and vegetable	1.275	1.250, 1.299	< 0.001*
**Greenspace exposure in quartiles (reference= Quartile 1)**			
quartile 2	0.921	0.898, 0.945	< 0.001*
quartile 3	0.934	0.910, 0.958	< 0.001*
quartile 4	1.003	0.977, 1.030	0.869

Blood pressure

Table 5: Single association between physical activity, fruit and vegetable intake, and greenspace exposure and blood pressure. Categories were Healthy, High (reference = Healthy).

Exposure variable (increment)	OR	95% CI	p adjusted
**Physical activity (reference= Healthy)**			
Moderate	0.979	0.947, 1.012	0.226
High	0.894	0.865, 0.924	< 0.001*
**Fruit and vegetable intake (reference= less than 5 portions)**			
High	0.960	0.935, 0.985	0.003*
**Greenspace exposure in quartiles (reference= Quartile 1)**			
quartile 2	1.093	1.055, 1.131	< 0.001*
quartile 3	1.133	1.094, 1.173	< 0.001*
quartile 4	1.069	1.031, 1.108	< 0.001*

Loneliness

Table 6: Single association between physical activity, fruit and vegetable intake, and greenspace exposure and loneliness. Categories were No, Yes (reference = No).

Exposure variable (increment)	OR	95% CI	p adjusted
**Physical activity (reference= Healthy)**			
Moderate	0.732	0.693, 0.773	< 0.001*
High	0.687	0.650, 0.726	< 0.001*
**Fruit and vegetable intake (reference= less than 5 portions)**			
High	0.876	0.836, 0.919	< 0.001*
**Greenspace exposure in quartiles (reference= Quartile 1)**			
quartile 2	0.979	0.924, 1.038	0.477
quartile 3	0.936	0.881, 0.994	0.036*
quartile 4	0.888	0.833, 0.945	< 0.001*

Model 2: Multi-exposure confounding model, no interactions between explanatory variables

Body Max Index (BMI)

Table 7: Multi-exposure association between physical activity, fruit and vegetable intake, and greenspace exposure and BMI, without interactions. Categories were Healthy, overweight, and Obese(reference = Healthy).

Exposure variable (increment)	OR	95% CI	p adjusted
**Physical activity (reference= Healthy)**			
Moderate	0.660	0.645, 0.676	< 0.001*
High	0.515	0.503, 0.527	< 0.001*
**Fruit and vegetable intake (reference= less than 5 portions)**			
High	1.110	1.089, 1.130	< 0.001*
**Greenspace exposure in quartiles (reference= Quartile 1)**			
Quartile 2	1.117	1.090, 1.144	< 0.001*
Quartile 3	1.136	1.109, 1.164	< 0.001*
Quartile 4	1.045	1.019, 1.071	0.001*

Overall health rating

Table 8: Multi-exposure association between physical activity, fruit and vegetable intake, and greenspace exposure and overall health rating, without interactions. Categories were Poor, Fair, Good, and Excellent (reference = Poor).

Exposure variable (increment)	OR	95% CI	p adjusted
**Physical activity (reference= Healthy)**			
Moderate	1.780	1.737, 1.824	< 0.001*
High	2.814	2.744, 2.886	< 0.001*
**Fruit and vegetable intake (reference= less than 5 portions)**			
High	1.155	1.133, 1.178	< 0.001*
**Greenspace exposure in quartiles (reference= Quartile 1)**			
quartile 2	0.936	0.912, 0.959	< 0.001*
quartile 3	0.949	0.925, 0.974	< 0.001*
quartile 4	1.025	0.999, 1.052	0.065

Blood pressure

Table 9: Multi-exposure association between physical activity, fruit and vegetable intake, and greenspace exposure and blood pressure, without interactions. Categories were Healthy, High (reference = Healthy).

Exposure variable (increment)	OR	95% CI	p adjusted
**Physical activity (reference= Healthy)**			
Moderate	0.983	0.951, 1.016	0.326
High	0.899	0.870, 0.930	< 0.001*
**Fruit and vegetable intake (reference= less than 5 portions)**			
High	0.973	0.947, 0.999	0.050*
**Greenspace exposure in quartiles (reference= Quartile 1)**			
quartile 2	1.091	1.054, 1.130	< 0.001*
quartile 3	1.132	1.093, 1.172	< 0.001*
quartile 4	1.067	1.029, 1.106	0.001*

Loneliness

Table 10: Multi-exposure association between physical activity, fruit and vegetable intake, and greenspace exposure and loneliness, without interactions. Categories were No, Yes (reference = No).

Exposure variable (increment)	OR	95% CI	p adjusted
**Physical activity (reference= Healthy)**			
Moderate	0.734	0.695, 0.775	< 0.001*
High	0.696	0.658, 0.735	< 0.001*
**Fruit and vegetable intake (reference= less than 5 portions)**			
High	0.904	0.861, 0.948	< 0.001*
**Greenspace exposure in quartiles (reference= Quartile 1)**			
quartile 2	0.971	0.916, 1.029	0.322
quartile 3	0.928	0.874, 0.986	0.018*
quartile 4	0.878	0.825, 0.936	< 0.001*
